# Integrated Analysis of the mTOR Signaling Pathway Mediated by the ORF3 Protein of Swine Hepatitis E Virus in HepG2 Cells via a circRNA–miRNA Network

**DOI:** 10.3390/vetsci13040350

**Published:** 2026-04-03

**Authors:** Jiya Li, Shengping Wu, Lingjie Wang, Xin Cao, Yulong Yin, Leli Wang, Hanwei Jiao

**Affiliations:** 1 College of Veterinary Medicine, Jilin Agricultural University, Changchun 130118, China; lijiya1125@outlook.com; 2 The College of Veterinary Medicine, Southwest University, Chongqing 402460, China; chemie@email.swu.edu.cn (S.W.); guolicheng666@email.swu.edu.cn (L.W.); 3 Institute of Subtropical Agriculture, Chinese Academy of Sciences, Changsha 410125, China; yinyulong@isa.ac.cn (Y.Y.); leliwang@isa.ac.cn (L.W.)

**Keywords:** swine hepatitis E virus, ORF3 protein, mTOR signaling pathway, circular RNA, HepG2 cells, m6A

## Abstract

Background: The ORF3 protein of swine hepatitis E virus (HEV-4) is a key virulence factor involved in viral assembly, egress, and host signaling regulation. The mammalian target of rapamycin (mTOR) pathway plays a pivotal role in autophagy, metabolism, and immunity, and is often modulated by viruses to promote replication. However, it remains unknown whether HEV-4 ORF3 modulates the mTOR pathway via circular RNAs (circRNAs). Methods: Using an adenovirus-mediated ORF3 overexpression system in HepG2 cells, we integrated circRNA and transcriptome high-throughput sequencing data, followed by KEGG enrichment analysis to identify mTOR-associated differentially expressed genes. A circRNA–miRNA regulatory network was constructed using bioinformatics tools, and the expression changes of m6A-related genes, including YTHDF3, were evaluated. Results: ORF3 overexpression significantly activated the mTOR pathway (*p* < 0.05) and led to the identification of 20 mTOR-related circRNAs (e.g., circRNA5142). These circRNAs regulated downstream autophagy and lipid metabolism genes by sponging miRNAs such as hsa-let-7d-5p and hsa-miR-132-3p. Altered YTHDF3 expression indicated possible m6A-dependent epitranscriptomic regulation of the mTOR pathway. Conclusions: Our integrated analysis suggests that HEV-4 ORF3 may modulate the mTOR pathway through a circRNA–miRNA network, perturbing host autophagy and metabolic balance, which may contribute to viral immune evasion. Targeting the ORF3-mediated circRNA-mTOR regulatory axis represents a promising therapeutic approach and provides a theoretical basis for novel anti-HEV-4 strategies.

## Simple Summary

This study suggests that HEV-4 ORF3 protein may modulate the host mTOR pathway by modulating circular RNAs, which sponge miRNAs to deregulate autophagy and lipid metabolism genes. The concurrent downregulation of the m6A reader YTHDF3 suggests epitranscriptomic involvement. This circRNA–miRNA network represents a novel mechanism for viral manipulation of host cell homeostasis, highlighting a potential therapeutic target.

## 1. Introduction

Hepatitis E virus (HEV) is an important zoonotic pathogen, the genotype of which—genotype 4 (HEV-4)—can cross species barriers to infect humans, utilizing the viral protein ORF3 to manipulate host cell processes, and it can be transmitted across species to humans, posing a significant threat to public health [1,2,3]. As a key virulence factor of HEV-4, the ORF3 protein is implicated in multiple stages of the viral life cycle, including replication and egress, and modulates host lipid metabolism. Notably, it can also interfere with host signaling pathways (e.g., the mTOR pathway) to promote viral immune evasion, as supported by recent evidence [4].

Circular RNAs (circRNAs) constitute a distinctive category of non-coding RNAs formed by back-splicing of precursor mRNAs, resulting in a covalently closed continuous loop that confers remarkable resistance to exonuclease-mediated degradation [5,6]. This structural stability underpins their function as competitive endogenous RNAs (ceRNAs). By sequestering microRNAs (miRNAs) via base-pairing, circRNAs act as molecular sponges, thereby attenuating the post-transcriptional repression of miRNA target genes. This interplay forms intricate circRNA-miRNA regulatory networks, which are now recognized as a pivotal layer of post-transcriptional gene regulation [7,8,9]. While such regulatory networks are known to be involved in regulating cellular autophagy, metabolism, and immune responses during viral infections, their precise functions in the context of HEV-4 infection are still elusive.

mTOR (mechanistic target of rapamycin) is a conserved serine/threonine kinase that forms mTORC1 and mTORC2 complexes, integrating nutrient, energy, and growth factor signals to regulate cell growth, proliferation, autophagy, metabolism, and protein synthesis [10,11]. Viruses such as HCV and HBV can promote their replication by modulating the mTOR pathway; however, whether HEV-4 ORF3 modulates the mTOR pathway via a circRNA-miRNA network remains to be explored [12,13].

Additionally, m6A RNA modification, as an epitranscriptomic regulatory mechanism, dynamically regulates mRNA stability, splicing, and translation through writers (e.g., METTL3/14), erasers (e.g., FTO, ALKBH5), and readers (e.g., the YTHDF family), playing key roles in viral infection. Studies indicate that m6A modification can influence mTOR pathway activity, but its function under the action of HEV-4 ORF3 remains unknown. Therefore, this study, based on the HepG2 cell model, aims to elucidate the mechanism by which HEV-4 ORF3 mediates the mTOR signaling pathway via a circRNA-miRNA network, providing novel insights into viral pathogenicity research. Therefore, this study aimed to preliminarily explore whether HEV-4 ORF3 may influence the mTOR signaling pathway through a circRNA-miRNA regulatory network by integrating circRNA and transcriptome sequencing data with bioinformatic predictions, thereby providing new hypotheses and clues for understanding its pathogenic mechanism.

## 2. Materials and Methods

### 2.1. Cell Model and Viral Protein Expression

HepG2 cells were purchased from the Cell Bank of the Chinese Academy of Sciences (purchased from the Shanghai Cell Bank of the Chinese Academy of Sciences, Shanghai, China). The HEV-4 ORF3 gene was overexpressed using an adenoviral vector (AD-ORF3), with AD-GFP serving as the negative control. The cells were cultured in DMEM medium supplemented with 10% fetal bovine serum. Cell samples were harvested 48 h post-infection. Successful ORF3 protein expression was verified via fluorescence microscopy and Western Blot analysis, as described previously.

### 2.2. High-Throughput Sequencing and Bioinformatics Analysis

Total RNA was isolated and subsequently subjected to circRNA and transcriptome sequencing, with libraries prepared from three independent biological replicates to ensure statistical robustness. Raw sequencing reads first underwent quality control and adapter trimming using Cutadapt (v2.1). The cleaned reads were then aligned to the human reference genome (Homo sapiens, Ensembl release-96) employing the splice-aware aligner HISAT2 (v2.2.1). Finally, to ensure comprehensive and accurate identification, circRNA prediction was performed by integrating the results from two independent algorithms: CIRCExplorer2 (v2.3.8) and CIRI2 (v2.0.6) [14,15]. To improve reliability, only circRNAs supported by at least two unique back-spliced reads and identified by both prediction tools were retained for subsequent analysis. Identification of differentially expressed genes (DEGs) was conducted using the edgeR package (v3.40.2). Transcripts meeting the thresholds of |log_2_(fold change)| ≥ 1.5 and a nominal *p*-value < 0.05 were classified as differentially expressed. To interpret the biological implications of the DEGs, we performed pathway enrichment analysis using the Kyoto Encyclopedia of Genes and Genomes (KEGG) database. Due to its established central role in regulating viral pathogenesis, cellular metabolism, and autophagy, the mTOR signaling pathway (KEGG entry ko04150) was prioritized for further in-depth analysis. Concurrently, m6A-related genes were screened from the list of differentially expressed mRNAs. This included writers (e.g., METTL3, METTL14, WTAP, VIRMA, RBM15/15B, ZC3H13), erasers (e.g., FTO, ALKBH5), and readers (e.g., YTHDF1/2/3, YTHDC1/2, IGF2BP1/2/3, HNRNPC, HNRNPA2B1). Their expression changes were subsequently analyzed.

### 2.3. circRNA-miRNA Network Prediction

To investigate the post-transcriptional regulatory potential of the differentially expressed circRNAs, a circRNA-miRNA interaction network was computationally constructed. The prediction workflow comprised two key steps. Initially, potential miRNA binding partners for the circRNAs were identified utilizing the TargetScan database (https://www.targetscan.org/; accessed on 16 March 2026), which specializes in predicting miRNA targets based on sequence complementarity. Subsequently, the mature sequence information and functional annotations for the predicted miRNAs were acquired from the miRBase repository (https://www.mirbase.org/; accessed on 16 March 2026). To ensure the reliability of the predicted interactions, a stringent filter was applied, retaining only those circRNA-miRNA pairs with a predicted binding site score greater than 0.9. These high-confidence interaction pairs were then integrated to build and graphically represent the circRNA-miRNA regulatory network.

## 3. Results

### 3.1. mTOR Signaling Pathway Identified by KEGG Enrichment Analysis

**Figure 1:**
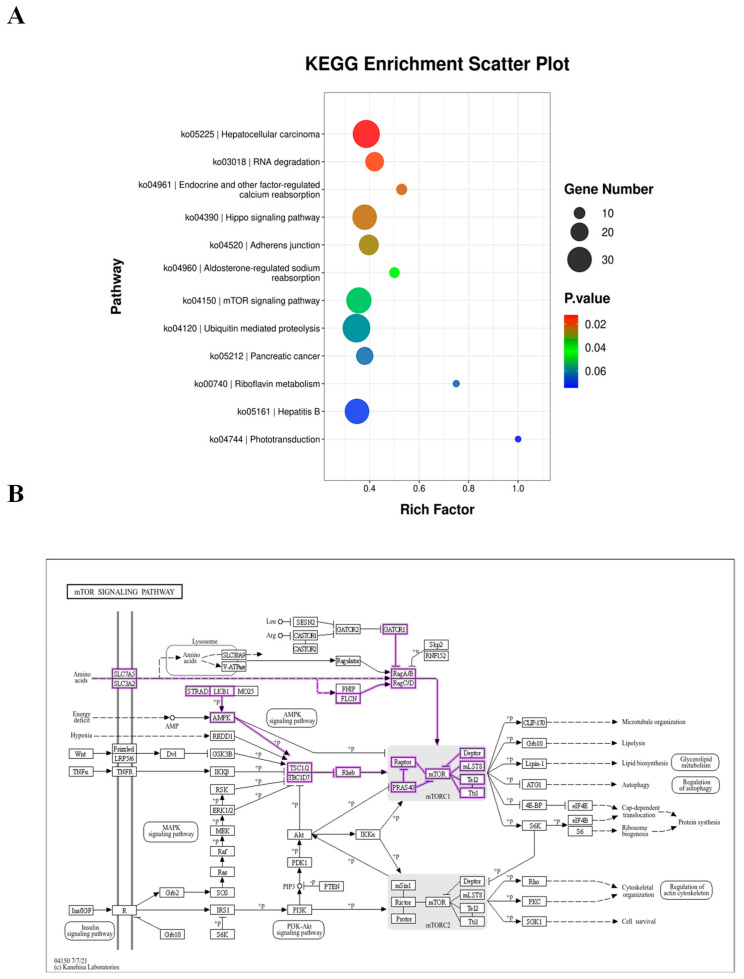
Enrichment analysis of the mTOR pathway in the context of HEV-4 ORF3 expression. (**A**): Visualization of KEGG pathway enrichment results, showing the top 12 significant pathways (*p* < 0.05); the mTOR pathway (ko04150) is indicated. (**B**): A graphical summary of the core components and interactions within the mTOR pathway.

KEGG enrichment analysis revealed that the mTOR signaling pathway (ko04150) was significantly enriched in the ORF3 overexpression group (*p* < 0.05) [16], ranking among the top 12 enriched pathways (Figure 1). This indicates that HEV-4 ORF3 may affect host cell function by regulating the mTOR pathway.

### 3.2. Screening of mTOR Pathway-Associated circRNAs

**Figure 2:**
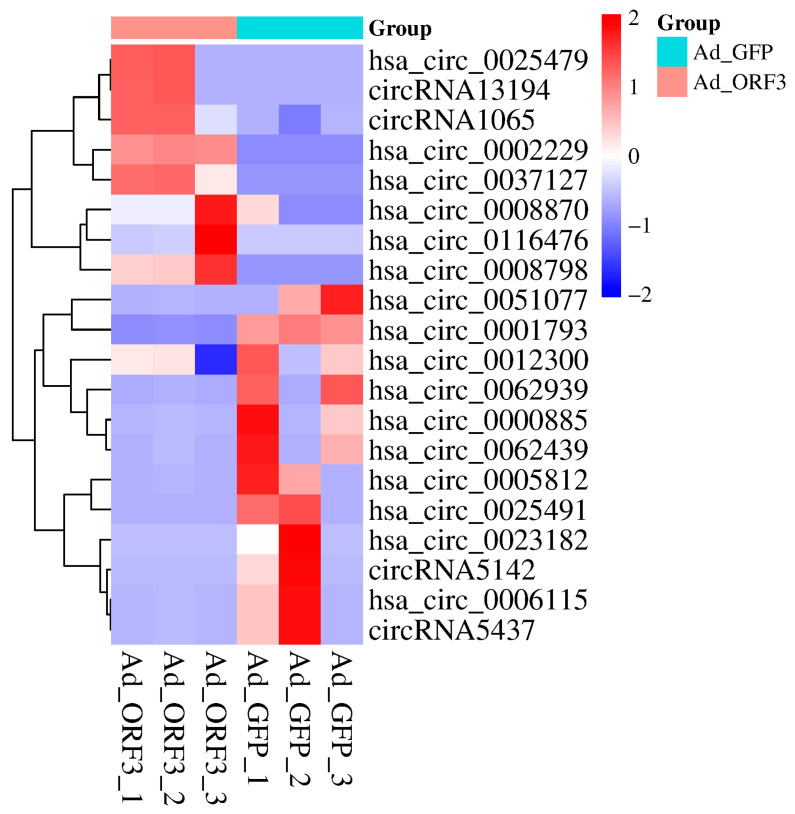
The heatmap depicts circRNAs within the mTOR pathway (ko04150) that were identified as statistically significant through transcriptome sequencing analysis.

Examination of the sequencing data revealed that 20 circRNAs linked to the mTOR signaling pathway were differentially expressed (Table 1). Among them, the expression of the following circRNAs was downregulated: circRNA5142 (ENSG00000177189), hsa_circ_0000885 (INSR), circRNA5437 (ENSG00000164327), hsa_circ_0025491 (LRP6), hsa_circ_0062939 (DEPDC5), hsa_circ_0023182 (LRP5), hsa_circ_0062439 (MAPK1), hsa_circ_0005812 (AKT2), hsa_circ_0006115 (PRKCA), hsa_circ_0051077 (AKT2), hsa_circ_0001793 (IKBKB), and hsa_circ_0012300 (PIK3R3). In contrast, the expression of the following circRNAs was upregulated: circRNA13194 (ATP6V1E1), hsa_circ_0002229 (LPIN1), hsa_circ_0037127 (NPRL3), hsa_circ_0025479 (LRP6), hsa_circ_0008870 (MAPK1), hsa_circ_0008798 (MAP2K1), circRNA1065 (RPS6KB1), and hsa_circ_0116476 (DEPDC5). Note that some circRNAs in Table 1 (e.g., circRNA5142) exhibited an extreme fold change (logged as ‘inf’ or ‘−inf’ due to near-zero expression in one group). Although the *p*-values for these specific cases were greater than 0.05, the large observed fold changes warranted their inclusion in the following exploratory network analysis. For all statistical reporting in this manuscript, a *p*-value below 0.05 is considered significant. The resultant expression patterns of these circRNAs are displayed in a clustered heatmap (Figure 2), which illustrates the variations across the different experimental samples.

### 3.3. circRNA-miRNA Regulatory Network Analysis

**Figure 3:**
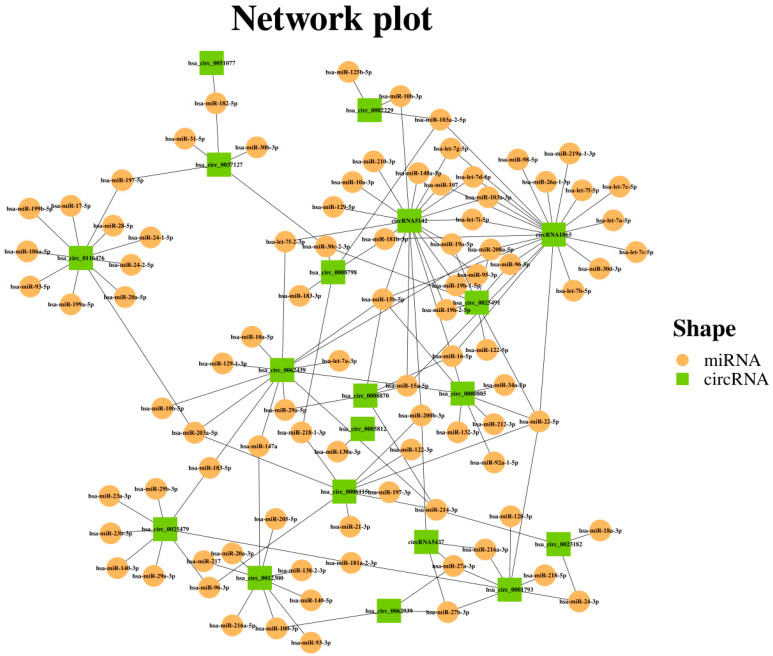
Within the validated mTOR pathway (ko04150), the target miRNAs of 20 circular RNAs (circRNAs) were predicted based on transcriptome data. These 20 validated circRNAs were used to construct a circRNA-miRNA network, where the target miRNAs are derived from transcriptome sequencing.

Prediction results indicate that the 20 circRNAs were predicted to target 146 miRNAs [17,18], forming a complex regulatory network (Figure 3). Among them, core circRNAs such as circRNA5142 and hsa_circ_0000885 can sequester miRNAs including hsa-let-7d-5p and hsa-miR-132-3p, which are known to modulate the mTOR pathway. This prediction suggests that HEV-4 ORF3 may influence autophagy and lipid metabolism via this potential network.

KEGG pathway enrichment analysis of the 20 mTOR-associated circRNAs revealed their potential involvement in a spectrum of biological processes beyond the mTOR pathway itself (Table 2). The most significantly enriched pathways included the core ‘mTOR signaling pathway’ (*p* = 0.052), as well as processes intimately linked to viral infection and the hypothesized mechanisms, such as ‘Autophagy—animal’, ‘Glycerolipid metabolism’, and ‘PPAR signaling pathway’. These pathways collectively suggest that the ORF3-mediated circRNA network may target not only the core mTOR pathway but also be broadly linked to biological processes such as cellular autophagy, lipid metabolism, and endoplasmic reticulum stress. This multi-pathway enrichment pattern implies that the ORF3 protein might coordinately regulate multiple interacting host cellular functional modules via this network, potentially creating an intracellular environment more favorable for viral persistence. Notably, pathways related to specific viral infections (e.g., ‘Hepatitis B’, ‘Human T-cell leukemia virus 1 infection’) and cellular stress responses (e.g., ‘Protein processing in endoplasmic reticulum’) were also prominently enriched, underscoring the potential broad impact of the ORF3-mediated circRNA network on host cell physiology.

### 3.4. Differential Expression Analysis of m6A-Associated Genes

To explore the potential impact of HEV-4 ORF3 on the m6A RNA modification machinery, we systematically screened for key m6A-related genes (writers, erasers, readers) from the list of differentially expressed mRNAs. The results showed that the expression levels of most m6A-related genes were not significantly altered. However, YTHDF3 expression was significantly downregulated (log_2_FC = −1.37, *p* < 0.05) (Table 3). Recent studies have shown that YTHDF3 enhances the translation efficiency of target mRNAs by recognizing m6A modifications, and its deficiency can disrupt mRNA metabolic balance [19,20]. For example, in glioblastoma, YTHDF3 cooperates with hnRNP A1 to promote IRES-mediated translation initiation, leading to sustained activation of the mTOR pathway. The downregulation of YTHDF3 suggests its potential involvement in modulating the metabolism of RNAs related to the mTOR pathway. However, this requires further experimental validation. KEGG enrichment analysis revealed that these m6A-related genes were not significantly enriched in specific pathways, such as the mTOR pathway (*p* > 0.05). However, as a core reader, YTHDF3 is known to regulate mRNA stability by recognizing m6A modifications, which may indirectly influence the expression of mTOR-associated genes.

## 4. Discussion

This integrated bioinformatic analysis reveals a potential association between HEV-4 ORF3 expression and the mTOR signaling pathway via a predicted circRNA-miRNA network. These findings are consistent with studies on riboflavin metabolism, indicating that viral proteins may modulate host metabolic pathways via non-coding RNAs (ncRNAs). The mTOR pathway, functioning as a cellular energy sensor, is crucial for maintaining metabolic homeostasis. Its dysregulation can lead to autophagy inhibition and lipid metabolism dysregulation [21]. This aligns with the hepatic injury observed in HEV-4 infection. Under physiological conditions, the mTOR pathway maintains cellular metabolic homeostasis. However, when dysregulated, it contributes to the pathogenesis of various major diseases.

The mTOR pathway plays a central role in cell growth, metabolism, and autophagy, and its dysregulation is implicated in various diseases, including cancer and autoimmune disorders [22,23,24,25,26,27,28]. This underscores the general significance of viral targeting of this pathway to disrupt host homeostasis and highlights the potential broader implications of HEV-4 ORF3-mediated mTOR dysregulation identified in this study.

It is important to note that the regulatory networks proposed herein are predicated on computational predictions and correlative analyses. To establish causal relationships and translate these associations into mechanistic understanding, rigorous experimental validation is imperative. Future work should encompass: (i) molecular validation using dual-luciferase reporter assays to confirm the direct binding between pivotal circRNAs (e.g., circRNA5142) and their predicted miRNA partners (e.g., hsa-let-7d-5p); and (ii) functional studies employing loss-of-function (e.g., CRISPR-Cas9 knockout, siRNA knockdown) or gain-of-function approaches to determine the consequent effects on mTOR pathway activity, autophagic flux, and lipid metabolic profiles in relevant cellular models. Furthermore, the association between the mTOR pathway and lipid metabolism suggests that HEV-4 may disrupt hepatocyte homeostasis via the ORF3-circRNA axis, providing a rationale for developing targeted therapies such as mTOR inhibitors.

Furthermore, this study is the first to reveal that overexpression of HEV-4 ORF3 leads to a significant downregulation of the m6A reader gene YTHDF3, suggesting that ORF3 may modulate the m6A modification machinery to regulate host gene expression [29]. Based on our findings and existing literature, we propose a testable working hypothesis: ORF3 may inhibit YTHDF3, leading to dysregulation of m6A modification, which could affect the stability of specific circRNAs (e.g., hsa_circ_0000885), thereby releasing their sponge effect on miRNAs (e.g., hsa-let-7d-5p) and ultimately alleviating the inhibition on mTOR pathway genes (e.g., AKT2) to promote viral immune evasion. This hypothesis is grounded in reports that YTHDF3 deficiency can enhance mTOR signaling activity [30], and that m6A-modified circRNAs can function as miRNA sponges [30]. Furthermore, a potential positive feedback loop is conceivable, as mTORC1 itself can promote m6A methylation by upregulating WTAP expression [31].

However, this inference requires further experimental validation, such as analyzing whether YTHDF3 target genes are enriched for mTOR pathway components via RIP-seq or dissecting the causal relationship using gene knockout models. Collectively, our integrated analysis not only posits a novel hypothesis wherein ORF3 modulates the mTOR pathway via a circRNA-ceRNA network but also, for the first time, links the downregulation of the m6A reader YTHDF3 to HEV-4 infection, offering a fresh epitranscriptomic perspective on its pathogenesis. From a translational standpoint, the identified ‘m6A-mTOR’ axis unveils a potential therapeutic vulnerability. Strategies aimed at disrupting this axis—for instance, by developing small-molecule inhibitors that interfere with the recognition of m6A-modified transcripts by YTHDF3—could evolve into a novel antiviral approach that hinders viral co-option of host resources [32], thereby delineating a new direction for future anti-HEV-4 drug development. Naturally, this hypothetical cascade involves multi-level regulation. Among these, the most critical and yet-to-be-verified links are whether the downregulation of YTHDF3 directly affects the stability or abundance of specific circRNAs (e.g., hsa_circ_0000885), and whether these circRNAs indeed function as effective molecular sponges for the predicted miRNAs.

Our KEGG analysis (Table 2) positioned the identified circRNAs within networks governing not only mTOR signaling and autophagy but also lipid metabolism (e.g., Glycerolipid and PPAR pathways) and ER stress. This convergence suggests that the HEV-4 ORF3 protein, via this circRNA-miRNA axis, may orchestrate a coordinated reprogramming of host cell metabolism and stress responses, creating a cellular environment favorable for viral replication and immune evasion. Theoretically, this coordinated reprogramming of metabolism and stress responses could be advantageous for the virus in achieving immune evasion, acquiring lipid precursors necessary for replication, and maintaining the survival of infected cells, although its specific functional impacts require experimental validation.

This study is primarily based on bioinformatic analysis and prediction, which presents certain limitations. First, the predicted circRNA-miRNA-mRNA regulatory networks require experimental validation through methods such as dual-luciferase reporter assays, RNA Pull-down, or RIP. Second, the causal relationship between YTHDF3 downregulation and mTOR pathway activation needs to be elucidated via gain- or loss-of-function experiments combined with functional rescue assays. Furthermore, the specific functional impact of this network on autophagy flux, lipid metabolism, and mTOR phosphorylation levels requires confirmation through subsequent cellular functional experiments. These will be the focus of our future research.

## 5. Conclusions

Through integrated analysis, we preliminarily identified an mTOR pathway-associated circRNA-miRNA regulatory network mediated by HEV-4 ORF3 and observed the downregulation of the m6A reader YTHDF3. These findings provide new insights into the pathogenesis of HEV-4 by proposing a novel hypothetical model in which the virus regulates host autophagy and metabolism via a circRNA-miRNA axis. This suggests that targeting the mTOR-circRNA network may represent a potential direction for developing antiviral strategies. Should the network predicted in this study be experimentally validated, interventional strategies could potentially include the direct use of mTOR pathway inhibitors or the development of molecular tools targeting core circRNAs (e.g., circRNA5142). However, the development of all such strategies is strictly dependent upon the stepwise functional confirmation of individual nodes within the network. Subsequent research will focus on functional validation and in-depth mechanistic studies to test this model.

## Figures and Tables

**Table 1:** List of differentially expressed circRNAs in the mTOR signaling pathway.

circRNA Gene ID	circRNA Gene Name	Log_2_ (Fold Change)	Regulation	*p*-Value	*q*-Value	Significant
circRNA5142	ENSG00000177189	−inf	down	0.13	1	yes
hsa_circ_0000885	INSR	−inf	down	0.13	1	yes
circRNA13194	ATP6V1E1	inf	up	0.15	1	yes
circRNA5437	ENSG00000164327	−inf	down	0.17	1	yes
hsa_circ_0002229	LPIN1	inf	up	0.18	1	yes
hsa_circ_0025491	LRP6	−inf	down	0.22	1	yes
hsa_circ_0062939	DEPDC5	−inf	down	0.22	1	yes
hsa_circ_0023182	LRP5	−inf	down	0.23	1	yes
hsa_circ_0062439	MAPK1	−inf	down	0.25	1	yes
hsa_circ_0005812	AKT2	−inf	down	0.26	1	yes
hsa_circ_0037127	NPRL3	inf	up	0.29	1	yes
hsa_circ_0025479	LRP6	inf	up	0.30	1	yes
hsa_circ_0008870	MAPK1	1.66	up	0.30	1	yes
hsa_circ_0006115	PRKCA	−inf	down	0.30	1	yes
hsa_circ_0051077	AKT2	−inf	down	0.30	1	yes
hsa_circ_0008798	MAP2K1	inf	up	0.31	1	yes
circRNA1065	RPS6KB1	1.88	up	0.33	1	yes
hsa_circ_0116476	DEPDC5	inf	up	0.34	1	yes
hsa_circ_0001793	IKBKB	−1.70	down	0.46	1	yes
hsa_circ_0012300	PIK3R3	−1.73	down	0.47	1	yes

**Table 2:** Top 10 Enriched KEGG Pathways for the Identified mTOR-Associated circRNAs.

KEGG ID	Pathway Name	Representative Enriched circRNAs	Gene Count	*p*-Value
ko04150	mTOR signaling pathway	circRNA5142, hsa_circ_0000885, hsa_circ_0005812 (AKT2)	31	0.0518
ko04140	Autophagy—animal	hsa_circ_0005812 (AKT2), hsa_circ_0008870 (MAPK1), hsa_circ_0012300 (PIK3R3)	24	0.5426
ko00561	Glycerolipid metabolism	hsa_circ_0002229 (LPIN1)	10	0.3695
ko03320	PPAR signaling pathway	hsa_circ_0023249 (CPT1A), hsa_circ_0063781 (PPARA)	10	0.3695
ko05166	Human T-cell leukemia virus 1 infection	hsa_circ_0001793 (IKBKB), hsa_circ_0005812 (AKT2), hsa_circ_0008870 (MAPK1)	35	0.2890
ko05161	Hepatitis B	hsa_circ_0005812 (AKT2), hsa_circ_0008870 (MAPK1), hsa_circ_0001793 (IKBKB)	30	0.0713
ko04146	Peroxisome	hsa_circ_0045746 (ACOX1), hsa_circ_0058450 (ACSL3)	12	0.2245
ko05230	Central carbon metabolism in cancer	hsa_circ_0005812 (AKT2), hsa_circ_0006006 (PDK1), hsa_circ_0006662 (GLS)	9	0.6078
ko04141	Protein processing in endoplasmic reticulum	hsa_circ_0002968 (MAPK8), hsa_circ_0045272 (ERN1)	38	0.1320
ko04152	AMPK signaling pathway	hsa_circ_0000885 (INSR), hsa_circ_0005812 (AKT2), hsa_circ_0008095 (RHEB)	23	0.2918

**Table 3:** Differential expression analysis of m6A-related genes.

Gene Id	Gene Name	log_2_(fc)	Regulation	*p*-Value	*q*-Value	Significant
ENSG00000145388	METTL14	0.24	up	0.41	1	no
ENSG00000165819	METTL3	−0.10	down	0.45	1	no
ENSG00000146457	WTAP	−0.04	down	0.71	1	no
ENSG00000255282	WTAPP1	−0.66	down	1	1	no
ENSG00000164944	VIRMA	0.15	up	0.38	1	no
ENSG00000259956	RBM15B	−0.10	down	0.45	1	no
ENSG00000162775	RBM15	0.02	up	0.99	1	no
ENSG00000123200	ZC3H13	−0.09	down	0.48	1	no
ENSG00000140718	FTO	0.21	up	0.27	1	no
ENSG00000091542	ALKBH5	0.02	up	0.99	1	no
ENSG00000185728	YTHDF3	−1.37	down	0.00	0.00	yes
ENSG00000149658	YTHDF1	0.08	up	0.65	1	no
ENSG00000198492	YTHDF2	−0.04	down	0.87	1	no
ENSG00000047188	YTHDC2	−0.15	down	0.29	1	no
ENSG00000083896	YTHDC1	0.10	up	0.58	1	no
ENSG00000159217	IGF2BP1	−0.08	down	0.44	1	no
ENSG00000136231	IGF2BP3	0.11	up	0.56	1	no
ENSG00000073792	IGF2BP2	0.06	up	0.75	1	no
ENSG00000092199	HNRNPC	−0.09	down	0.44	1	no
ENSG00000122566	HNRNPA2B1	−0.21	down	0.08	0.90	no

## Data Availability

The original contributions of this study are included in the article. Further inquiries can be directed to the corresponding authors.
